# Prenatal Diagnosis and Outcome of Umbilical–Portal–Systemic Venous Shunts: Experience of a Tertiary Center and Proposal for a New Complex Type

**DOI:** 10.3390/diagnostics12040873

**Published:** 2022-03-31

**Authors:** Rodica Daniela Nagy, Dominic Gabriel Iliescu

**Affiliations:** 1 Doctoral School, University of Medicine and Pharmacy of Craiova, 200349 Craiova, Romania; 2 Department of Obstetrics and Gynecology, University Emergency County Hospital, 200642 Craiova, Romania; dominic.iliescu@yahoo.com; 3 Department of Obstetrics and Gynecology, University of Medicine and Pharmacy of Craiova, 200349 Craiova, Romania; 4 Ginecho Clinic, Medgin SRL, 200355 Craiova, Romania

**Keywords:** umbilical–portal–systemic venous shunt, umbilical drainage, agenesis of ductus venosus, fetal venous shunt, venous anomalies, portal system, prenatal diagnosis, color Doppler

## Abstract

**Aims**: To share our experience in the prenatal diagnosis of umbilical–portal–systemic venous shunts (UPSVS) and to study the prognostic factors for proper prenatal and perinatal management. **Material and Methods**: A five-year prospective study regarding the detection of UPSVS was conducted in two referral centers, Medgin Ginecho Clinic and the Prenatal Diagnostic Unit of the tertiary center, University Emergency County Hospital Craiova, Romania. We included in the analysis a series of agenesis of ductus venosus (ADV) cases previously reported by our center. We analyzed the incidence of the UPSVS types, their associations, and outcome predictors. **Results**: UPSVS were diagnosed in all 16 cases that were presented to our center at the time of first trimester anomaly scan, except one (94.12%). We diagnosed: 19 type I (61.2%), 4 type II (12.9%) and 5 type IIIa (16.1%) UPSVS. In three cases (9.6%) we noted multiple shunts, which we referred to as type IV (a new UPSVS type). Type IIIa-associated fetal growth restriction (FGR) was found in 60% of cases. Major anomalies worsened the outcome. Of the UPVSS cases, 57.1% were associated with PVS anomalies. Genetic anomalies were present in 40% of the tested cases. **Conclusions**: The incidence of UPSVS in our study was 0.2%. Early detection is feasible. The postnatal outcome mainly depends on the presence of structural, genetic and PVS anomalies. FGR may be associated. The new category presented a poor outcome secondary to poor hemodynamic and major associated anomalies.

## 1. Introduction

Umbilical–portal–systemic venous shunts (UPSVS) occur due to the abnormal development or involution of the fetal vascular system during embryogenesis [1,2]. Various outcomes of these vascular anomalies have been reported, and most of the existing information comes from pediatric and adult studies. Patients with UPSVS may be asymptomatic postnatal, but they have a higher risk of two significant complications: the development of intrahepatic tumors (benign or malignant) and hepatic encephalopathy [3,4,5]. Other manifestations include hyperinsulinism and hyperandrogenism, secondary to a type I shunt, and hepatopulmonary syndrome, secondary to a type II shunt [6,7,8]. Postnatal cases may be discovered incidentally during the systematic screening for galactosemia performed in some countries [9,10]. Recent developments in ultrasonographic imaging have facilitated the prenatal diagnosis of fetal malformations. However, most of the cases have been reported as small series or isolated as part of the diagnosis of other fetal anomalies, because of the rarity of this condition [11,12,13,14,15,16]. Detailed investigation of fetal growth restriction (FGR) or cardiac anomalies increases the rate of UPSVS diagnosis [17]. The reviewed literature supports that these two anomalies are most common in UPSVS cases, besides genetic anomalies (Trisomy 21, Turner and Noonan syndromes), other vascular anomalies, congenital intestinal abnormalities, and kidney malformations [18]. The most frequent complication of the shunts is poor antenatal hemodynamic tolerance (dilated cardiomyopathy, pericardial effusion, and tricuspid insufficiency) [18].

Given the prenatal and postnatal implications, we believe that this condition, although rare, is very important. The study of the associations and outcomes would offer essential information to healthcare providers involved in prenatal diagnosis, and is of paramount importance for the counseling of parents.

The fetal circulatory system works differently from that in postnatal life and has several lost features at birth. The fetus is entirely dependent on placental circulation. The umbilical and left portal veins and DV form a unique fetal anatomical structure that allows the oxygen-rich blood from the placenta to reach the heart. The abnormalities of these three venous compartments may cause shunting to the systemic vessels [2,19]. These malformations have been classified under the term “umbilical–portal–systemic venous shunts” [20]. The concomitant chromosomal, cardiac, and portal venous system (PVS) abnormalities influence the outcome of prenatally detected UPSVS cases and direct perinatal management [21].

The purpose of our research was to present our experience in diagnosing and managing UPVS cases, concerning their associations and outcomes.

## 2. Materials and Methods

### 2.1. Study Design and Setting

The current prospective study was conducted in two referral centers, Medgin Ginecho Clinic and the Prenatal Diagnostic Unit of the tertiary center, University Emergency County Hospital Craiova, Romania, between January 2017 and December 2021. We included in the analysis a series of agenesis of ductus venosus (ADV) cases previously reported by our center between 2010–2013. The study aimed to analyze the incidence of the UPSVS types, their associations, and outcome predictors.

### 2.2. Participants

We included in the study, consecutively, all pregnancies that presented to our center for anomaly scan evaluations. Certified sonographers performed ultrasound (US) examinations with a minimum of three years of experience in obstetrical ultrasound. Ethical approval for the study was obtained from our institutional Ethics Committee and written informed consent was mandatory before ultrasound examination. The maternal characteristics, medical history, and associated fetal anomalies were noted.

### 2.3. Variables

**Figure 1:**
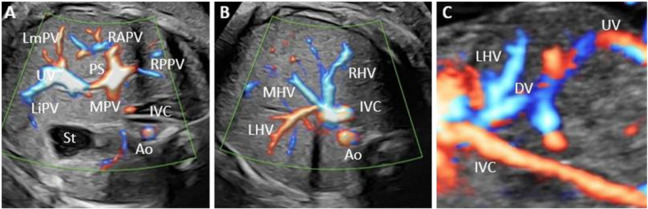
The typical aspect of the portal venous system (PVS), hepatic veins, and ductus venosus. (**A**) Transverse plane of the fetal abdomen, with high-definition directional power Doppler, applied to demonstrate the normal L-shaped UV confluence and PVS features. (**B**) Transverse plane allowed the evaluation of the left middle and right hepatic veins, aorta, and inferior vena cava. (**C**) Sagittal plane of the fetal thorax and abdomen, with the evaluation of the umbilical vein, left hepatic vein, ductus venosus, and inferior vena cava. UV, umbilical vein; RAPV, anterior branch of the right portal vein; RPPV, posterior branch of the right portal vein; LiPV, left portal vein inferior branch; LmPV, left portal vein medial branch; Ao, aorta; RHV, right hepatic vein; LHV, left hepatic vein; MHV, middle hepatic vein.

The primary variable was the umbilical–portal–DV complex normality, determined by the ultrasound identification of its features. Regardless of the age of the pregnancy, we evaluated the umbilical vein course and the presence of DV and inferior vena cava (IVC) (Figure 1). PVS evaluation was performed depending on the age of the pregnancy. We aimed to identify the umbilical vein (UV) and L-shaped portal sinus in the abdominal transverse plane during the first-trimester standard scan. We routinely searched for the portal confluent during the second and third trimesters, identifying the main portal vein, left portal vein, and anterior and posterior branches of the right portal vein.

Furthermore, when anomalies were suspected, we investigated the entire umbilical–portal–DV complex at any gestational age, following the three planes sweep, as described by Yagel et al. [22,23], and using the transvaginal route in the first trimester cases. In addition, the upper fetal abdomen’s ventral or lateral transverse planes allowed the supplementary evaluation of the left, middle, and right hepatic veins. When DV was absent, the type of the shunt was investigated. According to the prenatal classification, the shunt types were reported: type I, umbilical–systemic shunt (USS), type II, ductus venosus–systemic shunt (DVSS) and type III, portal–systemic shunt, divided into type IIIa, intrahepatic portal–systemic shunt (IHPSS) and type IIIb, extrahepatic portal–systemic shunt (EHPSS) [21]. We defined a new category, type IV, because we encountered new features of UPSVS. In this category we included multiple shunts, of different types. This finding might be of use in real-world practice, and thus the present data are unique and important.

We carefully analyzed the anatomic features of UPSVS-diagnosed fetuses. Additional sonographic findings and the outcome of the pregnancy were noted.

### 2.4. Methods of Assessment

According to the international guidelines, the transverse and longitudinal abdominal planes were examined [24,25]. In addition, a systematic color Doppler assessment of the abdominal venous system was proposed to all pregnant women who were admitted to our center. Color Doppler assessment was performed following the ALARA principle. Ultrasound (US) examinations were performed transabdominally and transvaginally using Voluson E10 and E8 (GE Medical Systems) ultrasound machines, equipped with a 4–8-MHz curvilinear and a RIC5-9-D transducer.

Regarding the timing of detection, we considered an abnormality to have been detected at the gestational age when the diagnosis was first suspected, even for cases where the diagnosis was confirmed at a later ultrasound scan.

Genetic counseling was performed in all cases, and the invasive investigation was proposed, including fluorescence in situ hybridization (FISH) and karyotype (standard), while the array comparative genomic hybridization (array-CGH) was reserved for selected cases. Detailed postnatal and follow-up evaluations at six months were performed in all UPSVS live births. Data were retrieved from the hospital database and by telephone interviews with the mothers. In addition, perinatal autopsy was offered in the cases that underwent pregnancy termination.

We also included in the current analysis the cases of agenesis of ductus venosus (ADV) previously reported between 2010 and 2013 in our center, when a similar study design was implemented for venous system assessment during the pregnancy anomaly scan [26]. The only difference in the designs of the two studies is that we did not aim to evaluate the portal system in the first trimester during the previous research, and a detailed examination was proposed at the beginning of the second trimester for ongoing pregnancies. The rationale to address the data acquired during the previous study was to extend the reported series and compare the incidence findings from the two time periods.

## 3. Results

A total of 31 cases with prenatally diagnosed UPSVS were identified in 14,793 fetuses with a prevalence of 0.209%. From the 31 cases, 11 (35.4%) were reviewed from the previously ADV reported cases, and 20 (64.5%) were diagnosed in the 7842 fetuses examined from 2017 to 2021. Given that the present study period was longer, the calculated prevalence was lower in the previous study (0.158%) than the present one (0.255%).

The gestational age at diagnosis ranged from 13 to 30 weeks. A total of sixteen cases were diagnosed at the time of FT anomaly scan: ten from the previous study, and all six presented FT cases from the current study. UPSVS were diagnosed in all cases evaluated in our center at the time of the first trimester (FT) anomaly scan, except one case from the previous study: isolated agenesis of ductus venosus (ADV), with a narrow umbilical–hepatic shunt, led to the typical aspect of ductal flow at the FT genetic scan. Thus, the anomaly was diagnosed during the second trimester. The 16 cases first evaluated in the FT are part of the 31 total cases presented in the study. The rest of the cases were first addressed at our center in the second or third trimester.

Genetic analysis was performed in 25 diagnosed cases, mainly by karyotyping, and chromosomal abnormalities were detected in 10 of them (40%): four (40%) Trisomies 21 (T21) (cases 3, 5, 7, 10), two (20%) Turner Syndromes (cases 1 and 29), two (20%) mosaicisms (cases 15 and 17), one (10%) translocation 46,XX,add(12)(q24) (case 9) and one (10%) 10p deletion (case 24).

We identified all classical types of shunts: type I, USS, 19 cases (61.2%); type II, DVSS, four cases (12.9%); and type IIIa, IHPSS, five cases (16.1%). We did not encounter type IIIb cases, EHPSS, but we identified three complex cases (9.6%) with multiple shunts. Therefore, we defined this category as type IV, complex variants with multiple shunts. From the 31 UPSVS cases, follow-up was possible in 29. The parents requested TOP in 12 cases (41.3%).

The prenatal findings and outcomes of the present 20 cases and 11 previously reported series are summarized in Table 1.

### 3.1. Umbilical–Systemic Shunts (USS, Type I)

**Figure 2:**
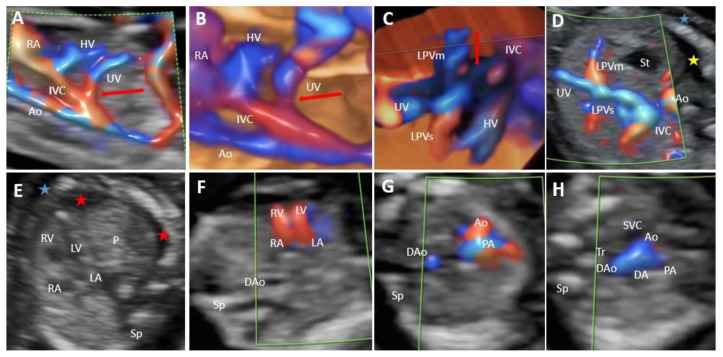
UPSVS type I with IVC drainage of the umbilical vein at 13 weeks of gestation (Case 1). (**A**) Color Doppler imaging in the longitudinal plane, showing the umbilical vein draining into the inferior vena cava. (**B**) Four-dimensional STIC reconstruction presenting the abnormal drainage of the umbilical vein (red arrow). (**C**) Four-dimensional STIC reconstruction, top view, showing the medial and superior branches of the left portal vein and the inferior vena cava drainage. (**D**) Axial view at the level of the fetal abdomen demonstrating the absence of the right portal vein and the umbilical vein connection to the inferior vena cava. Blue mark—skin edema, yellow mark—ascites. (**E**) Two-dimensional axial view at the thorax level showing the right-sided heart due to the presence of the pleural effusion (red mark). (**F**) Four-chamber view. During diastole, atrioventricular flows are visualized, separated by the interatrial and interventricular septum. (**G**) Color Doppler imaging, showing the pulmonary artery crossing the aorta in the axial plane. (**H**) Three vessels and trachea view showing the superior vena cava, pulmonary artery, ductus arteriosus, transverse aortic arch, and trachea. SVC, superior vena cava; Ao, aorta; PA, pulmonary artery; DA, ductus arteriosus; Dao, descending aorta; Tr, trachea. IVC, inferior vena cava; Sp, spine; RA, right atrium; LA, left atrium; RV, right ventricle; LV, left ventricle; P, lungs; UV, umbilical vein; HV, hepatic vein; Ao, aorta; LPVs, the superior branch of the left portal vein; LPVm, medial branch of the left portal vein; St, stomach.

**Figure 3:**
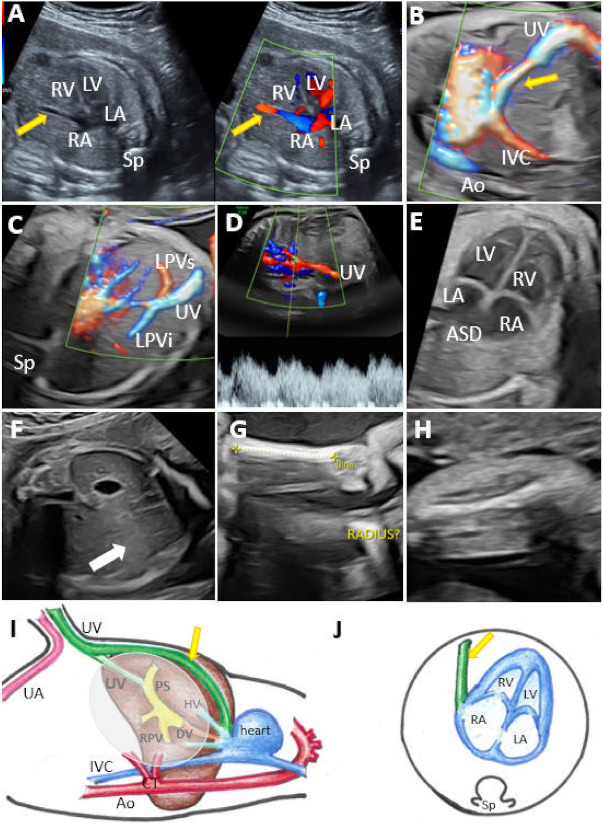
UPSVS type I with cardiac drainage of the umbilical vein at 24 weeks of gestation (Case 3). (**A**) Umbilical drainage (yellow arrow) directly to the right atrium visible in an axial–oblique plane of the upper abdomen and low thorax region, using duplex (color Doppler and gray-scale) imaging. (**B**) Directional power Doppler applied in the sagittal plane of the upper abdomen and low thorax region, showing the umbilical vein abnormal connection to the right atrium and absence of the ductus venosus. (**C**) Oblique plane at the abdomen level showing the absence of right portal vein. (**D**) Typical triphasic aspect of the shunt. (**E**) Four-chamber view, which demonstrates the absence of septum secundum. (**F**) The axial abdominal sweep could not find the gallbladder (white arrow). (**G**) Two-dimensional evaluation showing unilateral absence of the radius. (**H**) Short humerus. (**I**,**J**) Schematic diagram of type I shunt. (**I**) Sagittal plane with normal course of umbilical–portal–DV complex and hepatic veins observed by the transparency of the oval and also the abnormal course of the umbilical vein draining into the right atrium. (**J**) Four-chamber view, which demonstrates the abnormal drainage of the umbilical vein. IVC, inferior vena cava; Sp, spine; RA, right atrium; LA, left atrium; RV, right ventricle; LV, left ventricle; LPVs, the superior branch of the left portal vein; LPVi, the inferior branch of the left portal vein; ASD, atrial septal defect; UV, umbilical vein; Ao, aorta; HV, hepatic vein; PS portal sinus; RPV, right portal vein; CT, celiac trunk; DV, ductus venosus; UA, umbilical artery.

In the group with type I UPSVS, the umbilical vein was connected to the systemic circulation. The drainage was into IVC in eight cases (Figure 2), into the hepatic vein (HV) in four cases, and into the heart in seven cases (Figure 3). All cases were associated with ADV. From the 19 cases, PVS was assessed in 16 cases. The remaining three cases belonged to the previous study that evaluated PVS at the beginning of the second trimester. Thus, early termination of the pregnancy did not allow the further evaluation of the PVS in the respective three cases. PVS anomalies were present in 14 cases (87.5%). We noted total portal venous system agenesis (TPVSA) in nine cases (64.2%) and partial portal venous system agenesis (PPVSA) in five cases (35.7%). Two cases (12.5%) presented a normal PVS. Associated anomalies were present in all type I shunt cases, and in 11 cases (57.8%), the couple requested termination of the pregnancy (TOP). Regardless of the cases where TOP was the couple’s choice, we noted a case with a good outcome at birth and at six months follow-up, with an associated minor anomaly (hydronephrosis). We noted intrauterine fetal death (IUFD) in three cases, one infant death and one case of growth and motor retardation at six months follow-up. In two cases, the follow-up was not possible as the patients did not further refer to our center. Overall, we report a poor outcome in 94% of the evaluated cases for this group with type I. Genetic analysis was performed in 15 cases, with eight abnormal results (53.3%). Four cases refused the genetic assessment.

### 3.2. Ductus Venosus–Systemic Shunts (DVSS, Type II)

**Figure 4:**
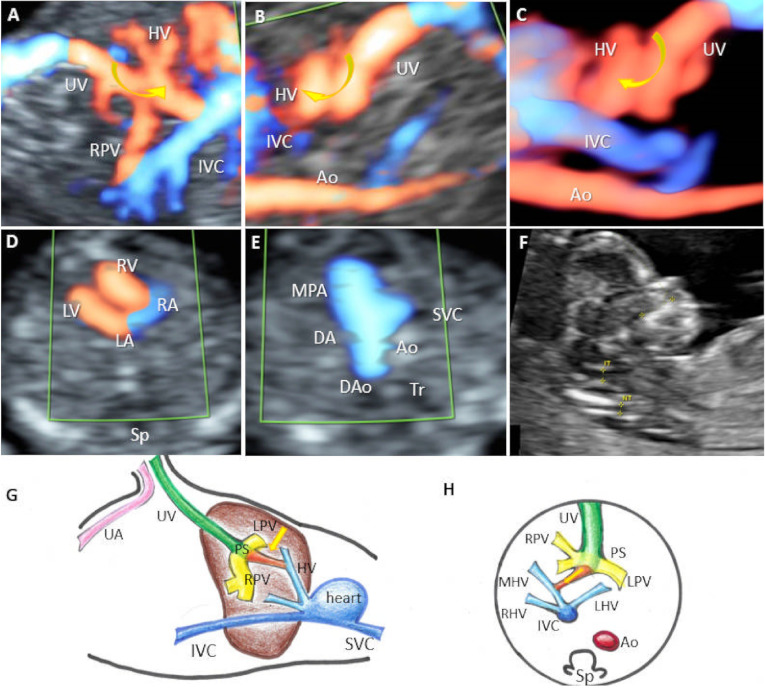
UPSVS type II with hepatic vein drainage of the umbilical vein at 13 weeks of gestation (Case 11). (**A**,**B**) In the sagittal plane, color Doppler imaging shows the umbilical vein draining through a short ductus venosus-like vessel (arrow) into the hepatic. (**C**) Four-dimensional STIC reconstruction, showing in the sagittal plane the abnormal drainage of the umbilical vein (blue circle). (**D**) Four-chamber view. During diastole, flow is visualized entering from the right and left atria (RA, LA) into the right and left ventricles (RV, LV), and the interatrial and interventricular septum separates the flows. (**E**) Normal “V-sign” with pulmonary artery, ductus arteriosus, and transverse aortic arch. (**F**) Midsagittal plane of the fetal head and upper thorax, showing normal appearance of the first-trimester genetic markers: nuchal translucency (NT) and nasal bone. (**G**,**H**) Schematic diagram of type II shunt. (**G**) Sagittal and (**H)** axial plane with abnormal course of umbilical vein draining into the hepatic vein through a ductus venosus-like vessel. SVC, superior vena cava; Ao, aorta; MPA, main pulmonary artery; DA, ductus arteriosus; Dao, descending aorta; Tr, trachea. IVC, inferior vena cava; Sp, spine; RA, right atrium; LA, left atrium; RV, right ventricle; LV, left ventricle; UV, umbilical vein; HV, hepatic vein; MHV, medial branch of the hepatic vein; RHV, right hepatic vein; LHV, left hepatic vein; Ao, aorta; RPV, right portal vein; LPV, left portal vein; PS portal sinus; RPV, right portal vein; DV, ductus venosus; UA, umbilical artery.

Four cases were classified as type II UPSVS (12.9%). A short ductus venosus-like vessel (DV) was present in all cases, but it drained into the HV in three cases (case 11) (Figure 4) and into the IVC in the remaining case. Normal PVS was present in all cases. The couple opted for pregnancy termination in one case, and three cases presented a good outcome at six months follow-up. A good outcome was observed in 75% of cases. In two cases, the couple refused the genetic evaluation, and the genetic results were normal for the other two cases.

### 3.3. Portal–Systemic Shunts (IHPSS, Type III)

Type IIIa UPSVS was detected in five patients (16.1%) (cases 15, 16, 17, 21, 22). In two cases (15 and 17) were associated the PPVSA, *FGR*, right aortic arch, duodenal atresia, and genetic anomalies (mosaicisms), which considerably influenced the postnatal outcome, with growth and motor retardation at six months follow-up.

**Figure 5:**
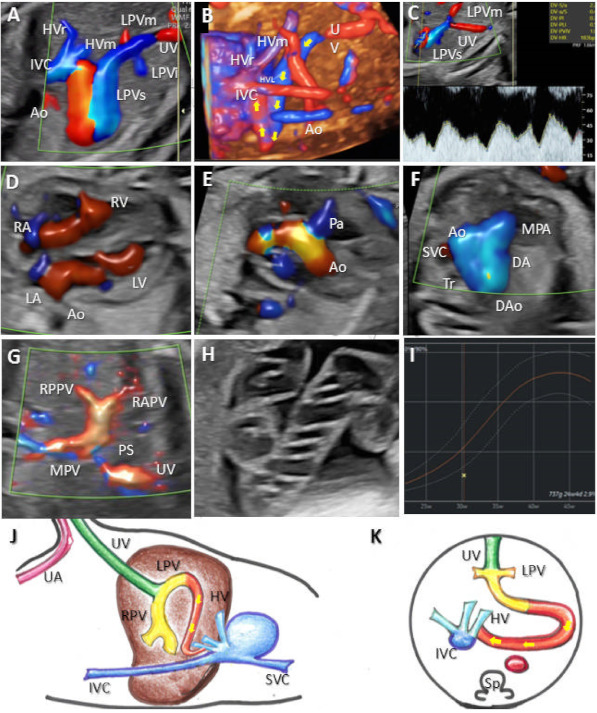
Type IIIa shunt with intrahepatic drainage of the umbilical vein at 30 weeks of gestation (Case 16) (**A**) Color Doppler imaging, showing in the oblique–transverse plane the connection between the superior branch of the left portal vein and the left hepatic vein (**B**) Four-dimensional STIC rendering of the abdominal vessels showing the pathway of the shunt. (**C**) Transverse view at the level of insertion of the umbilical cord showing intrahepatic drainage of the shunt and the biphasic aspect of the velocity. (**D**) Four-chamber view color Doppler assessment with flows separated by the interatrial and interventricular septum. (**E**) Color Doppler imaging, showing the left ventricular outflow tract in the axial plane. (**F**) Three vessels and trachea view showing the superior vena cava, pulmonary artery, ductus arteriosus, transverse aortic arch (from the proximal aorta to the descending aorta), and trachea. (**G**) Transverse plane of the fetal abdomen showing the typical UV confluence and portal venous system features. (**H**) Two-dimensional imaging of the hyper-coiled umbilical cord. (**I**) Graph of the fetal weight demonstrating a low percentile for the gestational age. (**J**,**K**) Schematic diagram of type III shunt. (**J**) Sagittal and (**K**) axial plane with abnormal connection between the superior branch of the left portal vein and the left hepatic vein. LPVi, the inferior branch of the left portal vein; LPVm, medial branch of the left portal vein; IVC, inferior vena cava; UV, umbilical vein; HVr, right branch of the hepatic vein; HVm, medial branch of the hepatic vein; HVl, left branch of the hepatic vein; Ao, aorta; Sp, spine.

Three cases presented a normal PVS and a good outcome. In case 16, an IHPSS was detected between the superior branch of the left portal vein and the left hepatic vein as a tortuous vessel (Figure 5). Further investigations showed normal genetic results, no additional morphological anomalies, and a good outcome at six months follow-up. The genetic evaluation was performed in all five cases, with mosaicism detected in two of them.

### 3.4. Complex Variants with Multiple Shunts (A Newly Described Category of UPSVS, Type IV)

**Figure 6:**
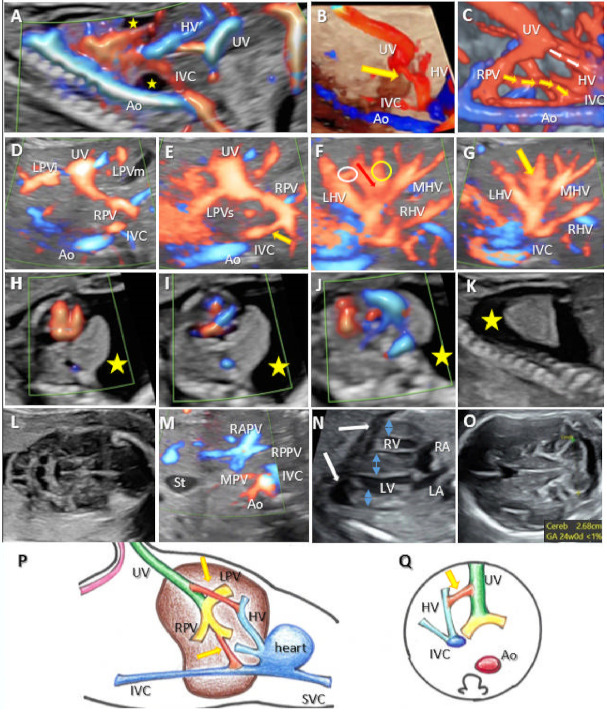
Complex drainage of the umbilical vein (UV) into the hepatic vein (HV) and two other intrahepatic shunts (one between the shunt and the left hepatic vein and the other between the right portal vein and the IVC) at 18 and 28 weeks of gestation (WG) (Case 18). (**A**) Color Doppler assessment at 18 WG showing the drainage of the UV into the middle branch of the HV. (**B**) Four-dimensional STIC rendering showing in the sagittal plane the short shunt vessel (yellow circle) connecting the umbilical and hepatic vein, below the prediaphragmatic infundibulum. (**C**) Four-dimensional STIC rendering showing two abnormal connections: one between the UV and the HV (white arrows) and another between the right portal vein and the IVC (yellow arrows). (**D**–**G**) Consecutive axial abdominal planes, part of the umbilical–portal and hepatic circulation investigation. (**D**) Caudally, a transverse plane at the level of the portal sinus. (**E**) A connection between the right portal vein and IVC (yellow arrow) is identified in the upper abdomen. (**F**) The white circle highlights a connection between the left hepatic vein and an abnormal shunt between one smaller venous branch of the portal vein (red arrow) and the umbilical vein. (**G**) Axial plane at the level of the hepatic efferent venous system and the umbilical–hepatic shunt (yellow circle). (**H**–**K**) The four-chamber and outflow tract views along with massive pleural effusion (yellow mark). (**L**) Ultrasound image of septated cystic hygroma at 18 WG. (**M**) Transverse plane at the level of the fetal abdomen with the typical aspect of the portal venous system. (**N**) Lateral view of the heart with hypertrophic ventricular walls and interventricular septum (blue arrows) and the presence of pleural effusion (white arrows). (**O**) Axial oblique posterior fossa view showing small cerebellar hemispheres. (**P**,**Q**) Schematic diagram of type IV shunt. (**P**) Sagittal and (**Q**) axial plane with abnormal course of umbilical vein draining into the hepatic vein and abnormal connection between right portal vein and inferior vena cava. RAPV, anterior branch of the right portal vein; RPPV, the posterior branch of the right portal vein; MPV, main portal vein; LPVs, the superior branch of the left portal vein. LPVi, the inferior branch of the left portal vein; LPVm, medial branch of the left portal vein; IVC, inferior vena cava; UV, umbilical vein; HV, hepatic vein; LHV, left branch of the hepatic vein; MHV, medial branch of the hepatic vein; RHV, right branch of the hepatic vein; LA, left atrium; LV, left ventricle; RA, right atrium; RV, right ventricle; St, stomach; Ao, aorta.

We diagnosed three cases of complex UPSVS (9.6%). PVS was found to be normal in all cases. In this group, minor malformations, such as a single umbilical artery (SUA) or non-visible gallbladder, were diagnosed in two out of three cases (66%). *FGR* was present in one out of the three cases (33%). Prenatal extensive genetic assessment (FISH, karyotype, array-CGH) was performed in all these cases and showed normal results. In case 20, the umbilical vein was connected to the IVC and the left HV. Motor retardation, coarctation of the aorta, and Kabuki Syndrome were detected postnatally. Coarctation of the aorta was confirmed postnatally. In case 19, the umbilical vein presented a complex intra- and extrahepatic drainage connected to the right HV and right atrium. IUFD occurred, possibly as a consequence of poor hemodynamics, including hydrops, pleural effusion, and ascites. Case 18 showed an abnormal vasculature due to abnormal drainage of the umbilical vein into the HV, and also two more atypical connections: one between the shunt and the left hepatic vein and the other between the right portal vein and the IVC (Figure 6, Video S1). In this case were associated hypertrophic cardiomegaly and pleurisy and neonatal death, signs of poor hemodynamics probably due to the presence of multiple abnormal connections. The genetic evaluation (only by the FISH assessment) showed normal results. The outcome of this case resulted in neonatal death.

### 3.5. Portal Venous System Characteristics in Relation to the Outcome

PVS was successfully evaluated in 28 UPVS cases. The early termination of pregnancy did not allow for a proper evaluation of the PVS in three cases. Out of the 28 cases, 16 cases (57.1%) were associated with PVS anomalies. In these cases, the poor prognosis was mainly due to the association of major abnormalities, which led to the choice of pregnancy termination in half of the cases (50%). The six-month follow-up was possible in three cases (18.7%) of those remaining, all associated with growth and motor retardation. Three IUFD cases (18.7%) were noted in this group, associated with hydrops in two of the cases. We lacked follow-up in two cases because the patients did not address our center for further evaluation and could not be contacted.

A normal PVS was demonstrated in 12 cases, seven of them (58.3%) with a good outcome at birth and at six months follow-up. An interesting case (case 16) showed a wide shunt between the superior branch of the left portal vein and the hepatic vein, no hemodynamic consequences, and a good outcome (Figure 5). Three cases had an apparently good outcome at birth, but significant structural and genetic-associated anomalies worsened the outcome (cases 18, 20, 24). The remaining two cases with a normal PVS had an unfavorable outcome: IUFD occurred in one case with complex drainage, which determined fetal hemodynamic alteration (case 19); TOP was the option chosen by the couple in the other case due to the association of hypoplastic left heart syndrome, asymmetric diaphragm and club foot (case 13).

## 4. Discussion

### 4.1. Epidemiological Considerations

The umbilical–portal–DV system represents a vascular unit, and any of its components may present abnormal connections [19]. We identified a total of 31 cases of UPSVS, and 96% were associated with other structural and/or genetic anomalies. Most of the prenatal literature consists of case reports. The UPSVS incidence in our study was 0.209%, which is higher than that previously reported: 1/30,000 live births (0.003%) [27], 0.016% [21], and 0.062% [9]. Still, we should note that our population was of medium risk as our tertiary center frequently receives referrals for fetal abnormalities. Of the 20 new cases, 8 were referred to our center.

According to the new in utero classification, from the three types previously reported, we managed to diagnose all of the UPSVS types, except type IIIb. Type I was the most frequently diagnosed (two-thirds of cases, 61.2%), which is in line with previous studies [21]. Multiple shunts of different types were present in three cases. Therefore, we could not place them in one of the categories previously described. Instead, we defined a new category of UPSVS when complex, multiple drainage types were diagnosed (Figure 6).

### 4.2. Early Detection

The feasibility of fetal PVS ultrasound evaluation at the FT anomaly scan has been previously demonstrated [28], as has the early detection of DV anomalies [26]. Our results confirm previous findings, as we managed to diagnose the shunt type in 16 of 17 cases (94.12%) who presented for the first time at the first-trimester anomaly scan.

### 4.3. The Outcomes of UPSVS Types—Associated Structural Anomalies

The association of major morphological and genetic anomalies worsened the outcome for type I (USS) cases. Anomalies of the PVS were present in 87.5% of the evaluated cases. Other associated anomalies were present in almost all (90%) of the cases, with a higher incidence than previously reported (55.6%) [20,21]. The type II (DVSS) group was characterized by a normal PVS in all four cases, with no structural anomalies in two cases and one non-lethal associated malformation. One major malformation was noted in the remaining case, and the couple underwent termination of the pregnancy due to the association of hypoplastic left heart syndrome. This group had the highest rate of a good outcome (75%). Type IIIa shunt (IHPSS) was associated with anomalies in 40% of cases. Interesting is that both of these cases were associated with duodenal atresia and right aortic arch. Despite the complex shunting pattern, type IV was characterized by a normal PVS, associated with heart defects and signs of poor fetal hemodynamics, which worsened the outcome in all cases. SUA was diagnosed in two out of the three cases (66.6%).

Associated anomalies are reported more often in cases of type I UPSVS (56%) and type IIIa (42%), while type II has the lowest incidence (15.8%) of associated anomalies [20]. Our data show a higher incidence of the major associated anomalies (90%) for type I UPSVS and type IIIa (40%), regardless of PVS anomalies. Instead, we found a lower incidence of major malformations in type II UPSVS (25%).

In our study the most frequently associated cardiac anomalies were hydrops and pleural effusion, signs of poor fetal hemodynamics. In addition, vascular, digestive, and reno-urinary malformations have been reported in the literature [18]. Such anomalies and others were found in our series: hydrops, duodenal atresia, esophageal atresia, right aortic arch, hydronephrosis, atrioventricular septal defect, heterotaxy syndrome, pulmonary atresia, hypoplastic left heart, univentricular heart, and holoprosencephaly. We also report the association of type I, TPVSA, T21, and aorto-umbilical fistulae in two cases. ADV was found in 83% of cases, and in the remaining cases, a short DV-like vessel was identified in type II shunts. Previous ADV studies also report this high association [29,30]. Thus, in line with the literature [20], we can support the idea that the absence of a normal DV should trigger a careful evaluation of the umbilical–portal system.

### 4.4. Portal Venous System Implications

Postnatal implications of PVS anomalies have been reported, mainly later in life, such as high blood galactose manifested in the neonatal period and, in adulthood, acute liver failure, portosystemic encephalopathy, or liver nodes [31,32,33,34,35]. More than half of the UPVSS cases (57.1%) were associated with PVS anomalies, whereas half of the cases were associated with a poor prognosis due to the association of major abnormalities. In addition, the apparently good prognosis at birth was hampered by growth and motor retardation at six months follow-up. A normal PVS was generally associated with a good outcome at birth and six months follow-up, especially if other structural or genetic abnormalities were not detected.

### 4.5. The Outcome of UPSVS Types—FGR

Our previous study cannot provide reliable data regarding the fetal growth pattern as the purpose of the second-trimester evaluation was to confirm the FT findings. Thus, the incidence of the FGR association was determined based on the new 20 cases.

Previous studies reported a high incidence of FGR in IHPSS cases (between 58% and 100%) [20,21,36]. Moreover, the detailed investigation of FGR fetuses can help diagnose IHPSS as this condition can alter fetal hemodynamics [37,38]. We also report a high incidence of this association for type IIIa, 60% of the cases. The reports suggest that the FGR may also occur in 39.5% of type I and II cases, regardless of the shunt type [20]. We cannot report an incidence of this association for type I and II because the potentially FGR cases could not be diagnosed due to early pregnancy termination or IUFD. In our view, in FGR cases, where chromosomal anomalies, infections, or placental insufficiency were ruled out, a careful evaluation of the portal venous system should be performed as half of the FGR cases presented PVS anomalies.

### 4.6. The Outcome of UPSVS Types—Genetics

In terms of invasive prenatal testing, controversial results were reported. For example, some studies showed a high rate of chromosomal abnormalities [21,39] (Trisomy 21, Turner and Noonan syndromes), but there are also reports which did not find a higher genetic pathology incidence among UPSVS cases (9%, regardless of the type of the shunt) [20]. In our study, an abnormal karyotype was found in 40% of the tested cases: four T21 cases (40%), two (20%) Turner Syndrome cases, two cases (20%) of mosaicism, one case (10%) of translocation 46, XX, add(12)(q24) and one case (10%) of 10p deletion. The prenatal genetic evaluation did not detect anomalies for the three type IV cases. However, the postnatal phenotype in case 20 required specific genetic tests, which detected Kabuki Syndrome, a rare multisystem disorder [40]. Heart abnormalities are the most commonly reported anomalies, and our case found that aortic coarctation was prenatally detected. Our study’s most frequently associated anomalies were cardiac anomalies, hydrops, and pleural effusion, as signs of poor fetal hemodynamic. Thus, we believe that a detailed structural and genetic evaluation is mandatory in UPSVS cases.

### 4.7. Complex Variants with Multiple Shunts

A previous publication of our group reported the possibility of a complex umbilical connection [41]. Although we did not find other confirmation of this finding in the literature, in our group, we identified three cases with multiple shunts. Case 18 (Figure 6) presented the umbilical vein draining into the middle branch of the hepatic vein, below the prediaphragmatic infundibulum. The careful sweep revealed abnormal connections between the left branch of the hepatic vein and the shunt and between the right portal vein and IVC despite the normal PVS appearance. The power Doppler interrogation of the shunt showed an atypical biphasic waveform, while previous reports indicate that the typical Doppler aspect of the shunts is triphasic at the point of drainage [20]. Contrary to our hypothesis based on previous findings, the normal results of extensive genetic assessment and the normal PVS did not improve the outcome of these cases; the associated major anomalies and complex vascular communications worsened the outcome. Still, the small number of detected cases does not allow us to draw safe conclusions regarding this condition. Counseling is often difficult in UPSVS cases, especially when rare variants are encountered.

UPSVS is a rare anomaly, and most of the prenatal studies consist of case reports, or it has been reported within the context of diagnosing ADV cases or fetal venous system anomalies [16,38,39,42]. Thus, we analyzed the previously published series, and we developed a design that would systematically evaluate the abdominal venous systems, including the PVS normalcy and the presence of the DV, regardless of the trimester of the pregnancy. Based on the present design, future research including more cases should be developed and the results added to current data, for a more accurate and informative analysis.

A limitation of our study was the unavailability of CGH array for all cases.

A strength of our study was that since the two study models were similar, we could present the results of a more extensive series of UPSVS with a standardized, systematic evaluation of the umbilical–portal venous system. The recognition and description of UPSVS types according to the new in utero classification allowed a better evaluation and characterization of the system, thus optimizing postnatal care. In addition, our study provides additional clinical insight into prenatal ultrasound by defining a new category of UPSVS—type IV shunt—which has not been previously defined.

## 5. Conclusions

The incidence of this pathology in a tertiary unit is higher than previously reported, at 0.2%. The early detection of UPSVS is feasible, which is important for proper management and prenatal counseling.

The outcome in UPSVS cases depends on the presence of concomitant structural or genetic abnormalities, which can occur in any type of UPSVS. Genetic assessment should be considered, as an abnormal karyotype was found in 40% of our cases. In addition, PVS assessment should be part of the evaluation, as the abnormalities of this venous system influence the postnatal outcome, even in the case of apparently good neonatal evaluation.

Care should be given to monitoring fetal wellbeing, as UPSVS can be associated with FGR. Additionally, FGR cases without an apparent cause should be investigated for UPSVS detection.

A new category of UPSVS was defined when complex, multiple drainage types were diagnosed. The newly defined category of UPSVS was associated with poor hemodynamics and significant malformations.

## Figures and Tables

**Table 1:** Sonographic findings and outcomes of 31 UPSVS cases.

Case	GA (Weeks)	Type of Shunt	Drainage	DV	PVS	Additional Sonographic Findings	Outcome	Intrauterine Genetic Assessment
Current study 2017–2022								
1	13	I	IVC	ADV	PPVSA	Hydrops	TOP	45X
2	13	I	IVC	ADV	TPVSA	-	TOP	N
3	24	I	Cardiac	ADV	PPVSA	Bilateral short humerus Unilateral absence of the radius Syndactyly Gallbladder agenesis ASD	NO FOLLOW-UP	T21
4	26	I	Cardiac	ADV	TPVSA	Persistent left superior vena cava esophageal atresia	Growth and motor retardation at 6 months	Invasive genetics declined
5	16	I	Cardiac	ADV	TPVSA	Hypoplastic left heart syndrome DORV; nasal hypoplasia	IUFD	T21
6	16	I	HV	ADV	PPVSA	-	NO FOLLOW-UP	Invasive genetics declined
7	15	I	HV	ADV	TPVSA	Aorto umbilical fistula Hygroma	TOP	T21
8	13	I	IVC	ADV	TPVSA	Hydrops	IUFD	Normal FISH
9	15	I	IVC	ADV	TPVSA	Hydrops Dextrocardia Aorto umbilical fistula Cheilognathopalatoschisis Ventriculomegaly	IUFD	Translocation 46,XX,add(12)(q24)
10	20	I	IVC	ADV	TPVSA	Aberrant right subclavian artery Small stomach	TOP	T 21
11	13	II	HV	Short DV-like	N	-	GAB Good at 6 months follow-up	Invasive genetics declined
12	20	II	HV	Short DV-like	N	Interrupted inferior vena cava with hemizygous continuation	GAB Good at 6 months follow-up	N
13	14	II	HV	Short DV-like	N	Hypoplastic left heart syndrome Asymmetric diaphragm Club foot	TOP	N
14	13	II	IVC	Short DV-like	N	-	GAB Good at 6 months follow-up	Invasive genetics declined
16	30	IIIa	HV	ADV	N	FGR Hyper-coiled umbilical cord	GAB Good at 6 months follow-up	N
17	28	IIIa	HV	ADV	PPVSA	FGR Duodenal atresia Esophageal atresia Right aortic arch	Growth and motor retardation at 6 months	mos 47, XY, +mar [15]/46, XY [38]
18	18	IV	Complex drainage HV + IVC	short DV	N	Pleural effusion Cardiomyopathy FGR Mitral and tricuspid regurgitation	GAB neonatal death	Normal FISH
19	13	IV	Complex HV + cardiac	ADV	N	Hygroma SUA Thymus agenesis Ascites Pleural effusion	IUFD	N
20	18	IV	Complex IVC + HV	ADV	N	SUA Non-visible gallbladder Coarctation of the aorta	GAB Kabuki Syndrome motor retardation at 6 months follow-up	N
The previous study, 2010–2013								
21	13^+6^	IIIa	HV	ADV	N	-	GAB Good at 6 months follow-up	N
22	13^+3^	IIIa	HV	ADV	N	-	GAB Good at 6 months follow-up	N
23	13	I	IVC	ADV	N	Hydronephrosis	GAB Good at 6 months follow-up	N
24	13	I	IVC	ADV	N	Cystic hygroma Abnormal facial profile-flat face Hypertelorism (ST findings) Skeletal: short femur and humerus, abnormal hands and feet (ST findings) Pyelectasis (ST finding) Choanal hypoplasia (neonatal finding) Restrictive respiratory disease	GAB Poor neonatal outcome Sudden infant death at 2 months	46 XY, 10p-
25	13^+5^	I	Cardiac	ADV	TPVSA	Cystic hygroma Renal pyelectasis Hypoplastic left heart	TOP	Invasive genetics declined
26	11	I	IVC	ADV	Not assessed (early termination)	Hydrops Atrio-ventricular septal defect Frontal bossing	TOP	N
27	13^+4^	I	Cardiac	ADV	TPVSA	Heterotaxy syndrome Pulmonary atresia Abnormal right subclavian artery Kyphoscoliosis	TOP ST	N
28	13^+2^	I	HV	ADV	PPVSA	Hydrops (ST finding) Hypoplastic left heart Single umbilical artery Short long bones (ST finding)	TOP ST	Invasive genetics declined
29	12^+6^	I	HV	ADV	PPVSA	Cystic hygroma Hydrops Hypoplastic left heart	TOP	45 X
30	11^+1^	I	Cardiac	ADV	Not assessed (early termination)	Hydrops Univentricular heart Abnormal face Absent NB Holoprosencephaly Microcephaly Skeletal abnormalities: spinal deformities, distorted feet, arm micromelia Lateral body wall defect Absent bladder	TOP	N
31	11^+3^	I	Cardiac	ADV	Not assessed (early termination)	Hydrops Body-stalk syndrome	TOP	N

GA, gestational age; DV, ductus venosus; HV, hepatic vein; IVC, inferior vena cava, PPVSA, partial portal venous system agenesis; TPVSA, total portal venous system agenesis; ASD, atrial septal defect; FGR, fetal growth restriction; TOP, termination of pregnancy; N, normal, FT, first trimester; IUFD, intrauterine fetal death; PVS, portal venous system; GAB, good at birth, NB, nasal bone, ST, second trimester; SUA, single umbilical artery; FISH, fluorescence in situ hybridization.

## Data Availability

Not applicable.

## Supplementary Materials

The following supporting information can be downloaded at: https://www.mdpi.com/article/10.3390/diagnostics12040873/s1, Video S1: (case 18). Type IV drainage.
